# Tick capillary feeding for the study of proteins involved in tick-pathogen interactions as potential antigens for the control of tick infestation and pathogen infection

**DOI:** 10.1186/1756-3305-7-42

**Published:** 2014-01-22

**Authors:** Sandra Antunes, Octavio Merino, Juan Mosqueda, Juan A Moreno-Cid, Lesley Bell-Sakyi, Rennos Fragkoudis, Sabine Weisheit, José M Pérez de la Lastra, Pilar Alberdi, Ana Domingos, José de la Fuente

**Affiliations:** 1 Instituto de Higiene e Medicina Tropical, Rua da Junqueira 100, 1349-008 Lisboa, Portugal; 2 SaBio. Instituto de Investigación en Recursos Cinegéticos IREC-CSIC-UCLM-JCCM, Ronda de Toledo s/n, 13005 Ciudad Real, Spain; 3 Facultad de Ciencias Naturales, Universidad Autónoma de Querétaro, Campus Juriquilla CP 76230, Querétaro Mexico; 4 The Roslin Institute, University of Edinburgh, Easter Bush, Midlothian EH25 9RG UK; 5 The Pirbright Institute, Ash Road, Pirbright, Woking GU24 0NF UK; 6 Centro de Malária e Outras Doenças Tropicais, Instituto de Higiene e Medicina Tropical, Rua da Junqueira 100 1349-008 Lisboa Portugal; 7 Department of Veterinary Pathobiology, Center for Veterinary Health Sciences, Oklahoma State University, Stillwater, OK 74078, USA

**Keywords:** Tick, Pathogen, *Anaplasma*, *Babesia*, Capillary feeding, Vaccine, Tick cell line

## Abstract

**Background:**

Ticks represent a significant health risk to animals and humans due to the variety of pathogens they can transmit during feeding. The traditional use of chemicals to control ticks has serious drawbacks, including the selection of acaricide-resistant ticks and environmental contamination with chemical residues. Vaccination with the tick midgut antigen BM86 was shown to be a good alternative for cattle tick control. However, results vary considerably between tick species and geographic location. Therefore, new antigens are required for the development of vaccines controlling both tick infestations and pathogen infection/transmission. Tick proteins involved in tick-pathogen interactions may provide good candidate protective antigens for these vaccines, but appropriate screening procedures are needed to select the best candidates.

**Methods:**

In this study, we selected proteins involved in tick-*Anaplasma* (Subolesin and SILK) and tick-*Babesia* (TROSPA) interactions and used *in vitro* capillary feeding to characterize their potential as antigens for the control of cattle tick infestations and infection with *Anaplasma marginale* and *Babesia bigemina*. Purified rabbit polyclonal antibodies were generated against recombinant SUB, SILK and TROSPA and added to uninfected or infected bovine blood to capillary-feed female *Rhipicephalus (Boophilus) microplus* ticks. Tick weight, oviposition and pathogen DNA levels were determined in treated and control ticks.

**Results:**

The specificity of purified rabbit polyclonal antibodies against tick recombinant proteins was confirmed by Western blot and against native proteins in tick cell lines and tick tissues using immunofluorescence. Capillary-fed ticks ingested antibodies added to the blood meal and the effect of these antibodies on tick weight and oviposition was shown. However, no effect was observed on pathogen DNA levels.

**Conclusions:**

These results highlighted the advantages and some of the disadvantages of *in vitro* tick capillary feeding for the characterization of candidate tick protective antigens. While an effect on tick weight and oviposition was observed, the effect on pathogen levels was not evident probably due to high tick-to-tick variations among other factors. Nevertheless, these results together with previous results of RNA interference functional studies suggest that these proteins are good candidate vaccine antigens for the control of *R. microplus* infestations and infection with *A. marginale* and *B. bigemina*.

## Background

Ticks are hematophagous ectoparasites whose feeding can have an adverse effect on animal health and production [1]. In particular, *Rhipicephalus* (*Boophilus*) spp. cattle ticks affect weight gain and milk production and transmit pathogens that cause bovine anaplasmosis (*Anaplasma marginale*) and babesiosis (*Babesia bigemina* and *Babesia bovis*) in tropical and subtropical regions of the world [2,3]. Chemical acaricides are currently the main method for the control of tick infestations, but problems associated with their use, such as limited efficacy in some regions due to selection of acaricide-resistant ticks and contamination of the environment and animal products with chemical residues, indicate the need for alternative control methods [4].

A promising alternative for the control of tick-borne diseases is vaccination with recombinant tick antigens [5]. Vaccination with commercial vaccines containing the recombinant *Rhipicephalus* (*Boophilus*) *microplus* BM86 gut antigen demonstrated their advantages for tick control, including cost-effectiveness and reduction in acaricide application. In addition, these vaccines also reduced the prevalence of anaplasmosis and babesiosis in some regions, presumably through reducing exposure of cattle to infected ticks [6]. However, BM86-based vaccines have variable efficacy against different geographic strains of *R. microplus* and do not affect tick vector capacity [6]. Therefore, new antigens are required for the development of vaccines affecting tick feeding, reproduction and vector capacity to control both tick infestations and pathogen infection/transmission.

Recently, the application of molecular biology approaches including transcriptomics and proteomics to the characterization of interactions between ticks (*Rhipicephalus* spp.) and pathogens (*A. marginale* or *B. bigemina*), with functional analyses using RNA interference (RNAi), have resulted in the identification of tick proteins with a possible role in pathogen infection and transmission [7-11]. However, additional analyses are required to characterize the potential of these proteins as vaccine antigens for the control of tick infestations and pathogen infection [12].

One approach to increase the possibilities of identifying tick protective antigens is to combine RNAi functional studies with *in vitro* tick feeding. RNAi allows screening of a relatively large number of genes involved in tick-pathogen interactions, while *in vitro* feeding with antibodies against selected candidate antigens should provide results more closely resembling vaccine protective capacity [12]. *In vitro* tick feeding techniques have been used for studies on tick biology and tick-pathogen interactions [13-19] and more recently to test the effect on tick feeding of antibodies added to the blood meal [20-22].

In the present study, we selected proteins involved in interactions between tick and *A. marginale* (Subolesin (SUB) and SILK) [7,9,10] and between tick and *B. bigemina* (TROSPA) [11], as determined by systems biology and RNAi functional studies, to characterize their potential as antigens for the control of both *R. microplus* tick infestations and infection with *A. marginale* or *B. bigemina* using *in vitro* capillary feeding with purified rabbit polyclonal antibodies against recombinant proteins. The results showed the possibilities and some of the limitations of this approach for the identification of candidate tick protective antigens.

## Methods

### Experimental design and rationale

The objective of this research was to evaluate the possibilities of identifying tick protective antigens using *in vitro* tick feeding with antibodies directed against tick proteins involved in tick-pathogen interactions, as determined by systems biology and RNAi functional studies. Antibodies were produced in rabbits using *R. microplus* recombinant proteins involved in tick-*A. marginale* (Subolesin (SUB) and SILK) [7,9,10] and tick-*B. bigemina* (TROSPA) [11] interactions. Partially engorged female *R. microplus* ticks were capillary fed with purified rabbit polyclonal antibodies against recombinant proteins added to blood collected from cattle uninfected and infected with *A. marginale* or *B. bigemina.* After *in vitro* capillary feeding, the effect of the treatments on tick weight, oviposition and pathogen DNA levels was measured as indicators of the potential of these antigens for the control of both *R. microplus* tick infestations and pathogen infection. Animal experiments were carried out in strict accordance with the Guide for Care and Use of Laboratory Animals for the University of Queretaro and the protocol was approved by the Committee on the Ethics of Animal Experiments (Permit no.: 23FCN2012).

### Expression and purification of recombinant proteins

The *R. microplus* SUB (Genbank accession number GQ456170.1) coding region was amplified by RT-PCR using oligonucleotides 5′-CACCATGGCGTGCGCCACCCTGAAAC-3′ and 5′-TTAAGACAGATAAGACGGGGTG-3′ and total RNA from the acaricide-susceptible and *Anaplasma-* and *Babesia*-free Media Joya strain, CENAPA, Mexico [23-25]. The genes encoding *Rhipicephalus* (*Boophilus*) *annulatus* TROSPA (JK489429) and *R. microplus* SILK (GO496219) proteins were synthesized by GenScript (Piscataway, NJ, USA). Tick genes were cloned into the expression vector pET101/D-TOPO® (Invitrogen, Carlsbad, CA, USA) following the manufacturer’s recommendations. Recombinant constructs were transformed into Bl21 *Escherichia coli* cells (Invitrogen, Carlsbad, CA, USA) and inoculated into Luria broth containing 50 mg/ml ampicillin and 0.4% glucose. Cultures were grown at 37°C to an OD_600nm_ = 0.4. Isopropyl-β-d-thiogalactopyranoside (IPTG) was then added to a final concentration of 0.5 mM and incubated for 4 h to induce the production of recombinant proteins. Cells were collected by centrifugation and recombinant proteins were purified to 80-90% purity by Ni affinity chromatography using the Ni-NTA Spin kit (QIAGEN, Hilden, Germany). Protein concentration was measured using the Pierce® BCA protein assay kit (Thermo Scientific, Rockford, IL, USA). Purified proteins were analyzed by SDS-PAGE and Western blot.

### Production of polyclonal antibodies

For each tick protein, three New Zealand white rabbits (*Oryctulagus cuniculus*) were subcutaneously injected at weeks 0, 3 and 6 with 50 μg protein in 0.5 ml Montanide ISA 50 V adjuvant (Seppic, Paris, France). Blood was collected before injection and two weeks after the last immunization to prepare preimmune and immune sera, respectively. Serum aliquots were kept at 4°C for immediate use or at −20°C for long-term storage. IgGs were purified from serum samples using the Montage Antibody purification kit and spin columns with PROSEP-A Media (Millipore, MA, USA) following the manufacturer’s recommendations.

### Western blot analysis

Ten micrograms of each protein were loaded into a 10% SDS-polyacrylamide pre-cast gel (Expedeon, San Diego, CA, USA). Samples were electrophoresed for 1 h at 180 V constant current. The SDS-PAGE gel was transferred to a nitrocellulose membrane (Whatman, Dassel, Germany) during 1 h at 12 V. The membrane was blocked with 5% skimmed milk in TBS for 1 h at room temperature, washed three times in TBS and incubated overnight with 2.5 μg/ml of the purified polyclonal IgGs in TBS, then washed three times with TBS and incubated with an anti-rabbit horseradish peroxidase (HRP) conjugate (Sigma-Aldrich, St. Louis, MO, USA) diluted 1:1000 in TBS. The membrane was washed three times with TBS and finally developed with 3, 3′, 5, 5′-tetramethylbenzidene (TMB) stabilized substrate for HRP (Promega, Madison WI, USA).

### Tick cell lines

Three embryo-derived tick cell lines, *R. microplus* BME/CTVM2 [26] and *Ixodes scapularis* IDE8 [27] and ISE6 [28] were used in the experiment. BME/CTVM2 cells were cultured in L-15 (Leibovitz) medium supplemented with 10% tryptose phosphate broth (TPB) and 20% fetal calf serum (FCS) at 28°C. IDE8 and ISE6 cells were grown in L-15B medium [29] supplemented with 10% TPB, 5% FCS and 0.1% bovine lipoprotein concentrate (MP Biomedicals, UK) at 32°C. Both media were supplemented with 2 mM L-glutamine, 100 IU/ml penicillin and 100 μg/ml streptomycin. Tick cell lines were maintained in flat-sided tubes (Nunc). Medium changes were carried out weekly by removal and replacement of two-thirds of the medium volume. Cultures were passaged at a split ratio of 1:1 at 2–3 week intervals as follows: an equal volume of fresh medium was added to the parent tube, cells were resuspended by gentle pipetting, and half the resultant cell suspension was transferred to a new culture tube (previously conditioned by incubating fresh culture medium therein for several hours) while leaving the remainder in the parent tube for reattachment.

### Immunofluorescence in tick cells

For the immunofluorescence assay, tick cells were harvested by pipetting, counted by haemocytometer and seeded at 5–6 × 10^5^ cells/ml in 1 ml fresh medium onto glass coverslips inside 24-well plates. After overnight incubation the cells were fixed *in situ* with 10% neutral buffered paraformaldehyde for 1 h. After a wash with PBS, the cells were permeabilized by covering with 0.3 ml Triton X-100/PBS for 30 min. The Triton X-100/PBS was removed and the cells were coated with 0.1% SDS/PBS for 10 min. The cells were then washed with PBS and blocked with CAS-block (Invitrogen, Paisley, UK) for 60 min and then incubated overnight at 4°C with the purified IgGs diluted 1:100 in CAS-block. After at least 3 × 5 min additional washes with PBS, they were incubated in CAS-block containing a secondary FITC-conjugated anti-rabbit antibody (1:500; AbD Serotec, Oxford, UK) for 1 h at room temperature. Cells were washed three more times with PBS and the coverslips were drained and mounted onto microscope slides using Vectashield hardset mounting medium (Vector Laboratories, Peterborough, UK). For fluorescence and light microscopy, a confocal microscope was used (Zeiss AxioSkop confocal microscope; Carl Zeiss Ltd., Welwyn Garden City, UK) and images were analyzed with Zeiss Zen software.

### Immunofluorescence in tick tissues

Tick guts, salivary glands and ovaries were dissected in ice-cold PBS from individual engorged *R. microplus* females. All tissues were washed in PBS and the luminal content was carefully removed from the guts. Tissues were either used immediately after dissection or stored at −80°C in RNAlater (Ambion, Austin, TX, USA) until use. To prepare samples for indirect fluorescence microscopy, dissected tissues were placed in small plastic cassettes, fixed in 3.7% formaldehyde, dehydrated in increasing ethanol dilutions and infiltrated with paraffin (Histosec, Merck, Whitehouse Station, NJ, USA). After cooling, tissues were sectioned using a SM2010 R sliding microtome (Leica, Carnaxide, Portugal). The 2–3 μm sections were placed on a glass slide, allowed to dry and then subjected to deparaffinization and dewaxing in xylene and hydrated in decreasing ethanol concentrations. Tissues were then permeabilized with 0.5% Triton X-100 (v/v) in PBS for 30 min and washed three times with excess PBS before blocking overnight with 3% BSA (w/v) at 4°C. After washing the slides again with PBS, solutions of purified IgGs diluted 1:100 in blocking solution were applied and incubated for 1 h at 37°C. The slides were then washed three times with PBS and the secondary antibody Alexa Fluor 488 (green)-conjugated anti-rabbit antibody (Molecular Probes, Life Technologies, Porto, Portugal) diluted 1:100 in blocking solution was applied and slides incubated for 1 h at 37°C. After a final PBS wash, a drop of ProLong® Gold Antifade Reagent with 4′,6′-diamidino-2-phenylindole (DAPI) (Invitrogen) was placed over the sections and then sealed with a coverslip. Slides were kept in a moist dark box until microscopic analysis to prevent drying and fluorescence fading. Tick tissue sections were visualized under a Nikon Eclipse 80*i* fluorescence microscope with appropriate filters (Nikon Instruments Inc., Barcelona, Spain).

### Tick capillary feeding

To obtain *B. bigemina* and *A. marginale* infected blood, two 6 month-old *Babesia/Anaplasma*-free calves were splenectomized and two weeks later intravenously inoculated one each with cryopreserved 1×10^6^*B. bigemina* (Chiapas strain; [30]) or 1×10^8^*A. marginale* (Morelos strain; [31]) infected erythrocytes (IE). After inoculation, animals were regularly monitored by checking their rectal temperature and hematocrit, and by examination of Giemsa-stained blood smears. Once the parasitemia levels reached 0.7% IE, 500 ml blood was collected into collection bags containing 70 ml citrate phosphate dextrose anticoagulant. Uninfected bovine blood was obtained in a similar manner from a healthy calf.

Tick artificial feeding was carried out with partially engorged female *R. microplus* (Media Joya strain) ticks recovered manually from calves 20–21 days after infestation with larvae. Collected ticks were cleaned, weighed and fixed on expandable polystyrene plates (19 × 10 cm) with double-sided adhesive tape (3 M, St. Paul, MN, USA). Female ticks were discarded if they had damaged mouthparts or their weight did not lie between 20 and 60 mg. Citrated bovine blood from uninfected or infected animals was used to fill microhematocrit capillary tubes (75 × Ø1.5 mm) that were placed over the ticks’ mouthparts. Tubes were replaced every 2–3 h, as described previously [22]. Female ticks were divided into experimental groups of 15 individuals each and fed for 28 h with uninfected, *B. bigemina*-infected or *A. marginale*-infected blood alone or supplemented with 1 mg/ml of preimmune or antigen-specific purified IgGs. After feeding, ticks were detached from the double-sided tape and weighed again to determine tick weight increase during feeding. Five ticks per group were then placed in Petri dishes and incubated at 27°C and 85% humidity for oviposition [7]. Weight increase during feeding (mg/tick) and oviposition (weight of eggs/tick in mg) were compared between ticks fed with blood supplemented with antibodies against the selected recombinant proteins and control ticks fed with bovine blood supplemented with preimmune antibodies by Student’s t test with unequal variance (P = 0.05).

This study was carried out in strict accordance with the Guide for Care and Use of Laboratory Animals for the University of Queretaro and the protocol was approved by the Committee on the Ethics of Animal Experiments (Permit no.: 23FCN2012).

### PCR to determine pathogen DNA levels in ticks

In order to quantify pathogen DNA levels, 10 ticks per group were incubated in Petri dishes at 27°C and 85% humidity for three days after feeding and their internal tissues dissected to obtain total RNA and DNA using TRI Reagent (Invitrogen, Carlsban, CA, USA) following the manufacturer’s recommendations. For each tick, *A. marginale* and *B. bigemina* DNA levels were analyzed by real-time PCR using the oligonucleotide primers RTMSP4F 5′-GACGTGCTGCACACAGATTT-3′/RTMSP4R 5′-CTCATCAAATAGCCCGTGGT-3′ and RTBbF 5′-AGCTTGCTTTCACAACTCGCC-3′/RTBbR 5′-TTGGTGCTTTGACCGACGACAT-3′ to amplify the *A. marginale* major surface protein 4 (*msp4*) (AF428083) and *B. bigemina* 16S rDNA (HQ264118) genes, respectively. Real-time PCR was performed using the QuantiTec SYBR Green RT-PCR kit (Qiagen, Valencia, CA, USA) and a Bio-Rad iQ5 thermal cycler following the manufacturer’s recommendations. The *A. marginale msp4* and *B. bigemina* 16S rDNA levels were normalized against tick 16S rDNA using the comparative Ct method [32]. Pathogen DNA levels in ticks (arbitrary units) were compared between ticks fed with blood supplemented with antibodies against the selected recombinant proteins and control ticks fed with bovine blood supplemented with preimmune antibodies by Student’s t test with unequal variance (P = 0.05).

### Characterization of tick mRNA levels of genes encoding for vaccine antigens

The *sub*, *silk* and *trospa* mRNA levels were characterized in 10 ticks per group by real-time RT-PCR [33] using the oligonucleotide primers SubolrtFw:5′-CACAGTCCGAGTGGCAGAT-3′ and SubolrtRev:5′-GATGCACTGGTGACGAGAGA-3′, SilkrtFw:5′-GGACGTCAATCCGCATTTGG-3′ and SilkrtRev:5′-GGCCATAGCCGTAGTTTCCA-3′, TROSPArtFw:5′-AGGTTACGGACACGGAGGA-3′ and TROSPArtRev:5′-GCCCAAGCGCATAAATAAGA-3′ for *sub*, *silk* and *trospa*, respectively. Real-time RT-PCR was performed using the QuantiTec SYBR Green RT-PCR kit (Qiagen, Valencia, CA, USA) and a Bio-Rad iQ5 thermal cycler (Hercules, CA, USA) following the manufacturer’s recommendations. The mRNA levels were normalized against tick 16S rRNA using the comparative Ct method [32]. Normalized mRNA levels were compared between ticks fed with blood supplemented with antibodies against the selected recombinant proteins and control ticks fed with bovine blood supplemented with preimmune antibodies by Student’s t test with unequal variance (P = 0.05).

## Results

### Production and characterization of antibodies against tick proteins involved in tick-pathogen interactions

**Figure 1:**
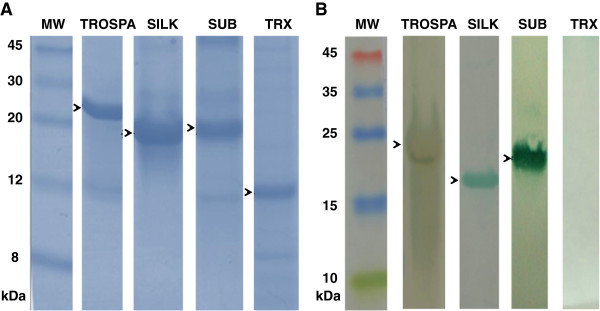
**Production of recombinant proteins and antibodies. (A)** Ten micrograms of recombinant proteins TROSPA, SILK, Subolesin (SUB) and thioredoxin (TRX; negative control) were loaded onto an SDS-PAGE gel under reducing conditions and visualized by Coomassie brilliant blue staining. **(B)** Western blot analysis of the recombinant proteins using purified IgGs produced in rabbits immunized with these proteins. Hybridization signals were developed with an anti-rabbit IgG HRP conjugate and TMB substrate for HRP. Arrows indicate the position of recombinant proteins. Abbreviation: MW, molecular weight.

Proteins involved in tick-*A. marginale* (Subolesin (SUB), SILK) and tick-*B. bigemina* (TROSPA) interactions as determined by systems biology and RNAi functional studies were selected to characterize their potential as antigens for the control of both *R. microplus* tick infestations and infection with *A. marginale* or *B. bigemina*. Recombinant proteins were produced in *E. coli* (Figure 1A) and used to obtain polyclonal antibodies in rabbits for *in vitro* capillary tick feeding experiments.

**Figure 2:**
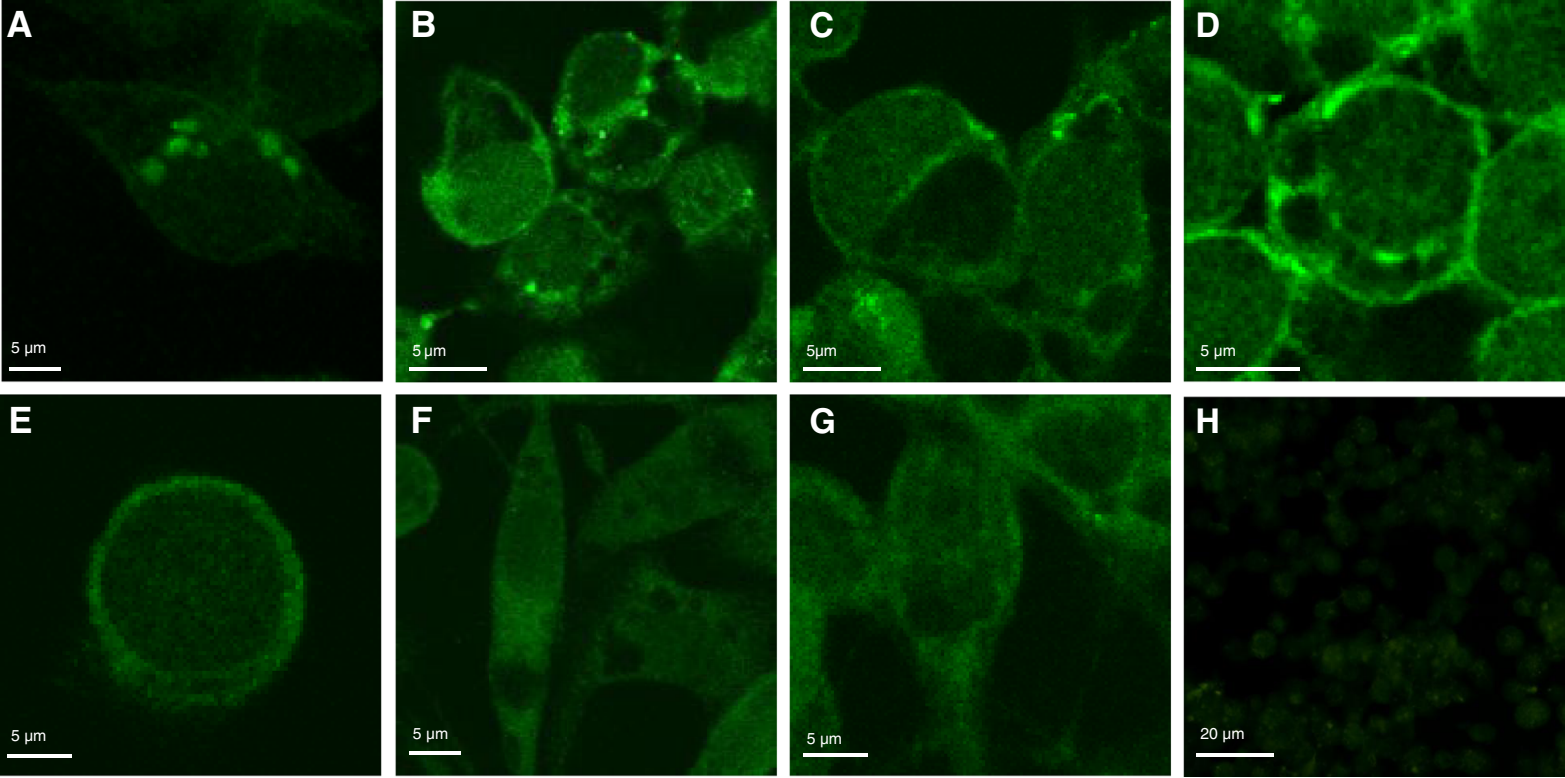
**Immunofluorescence analysis of tick cells.** Representative images of imunofluorescence analysis of tick cells. Tick cells were stained with rabbit anti-tick protein antibodies (green, FITC). **(A, B)** BME/CTVM2 cells stained with anti-SILK antibodies. **(C, D)** BME/CTVM2 cells stained with anti-TROSPA antibodies. **(E)** BME/CTVM2 cells stained with anti-SUB antibodies. **(F)** IDE8 cells stained with anti-SUB antibodies. **(G)** ISE6 cells stained with anti-SUB antibodies. **(H)** preimmune control serum-treated BME/CTVM2 cells. Scale bars, 5 μm **(A-G)**; 20 μm **(H)**.

**Figure 3:**
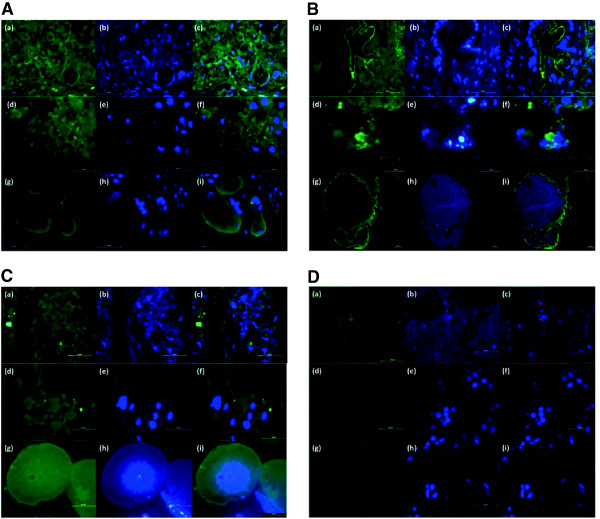
**Immunofluorescence analysis of tick tissues.** Representative images of imunofluorescence analysis of *R. microplus* engorged female tick tissues. Tick salivary gland (a-c), gut (d-f) and ovary (g-i) tissue sections were stained with rabbit anti-tick protein antibodies (green, Alexa Green 488; (a, d, g)) and DNA was stained with DAPI (blue; (b, e, h)). Merged images are also shown (c, f, i). **(A)** Tick tissue sections were stained with anti-SILK antibodies. **(B)** Tick tissue sections were stained with anti-TROSPA antibodies. **(C)** Tick tissue sections were stained with anti-SUB antibodies. **(D)** Tick tissue sections were stained with preimmune control serum. Scale bars, 50 μm.

The antibodies were first characterized by Western blot showing the specific recognition of their respective denatured antigens (Figure 1B). The antibodies were then characterized for their ability to recognize tick proteins by immunofluorescence in cultured tick cells (Figure 2A-H) derived from both homologous (*R. microplus*) (Figure 2A-E) and heterologous (*I. scapularis*) (Figure 2F and G) species and in *R. microplus* fed female guts, salivary glands and ovaries (Figure 3A-D). Preliminary analysis showed that all antibodies recognized tick proteins in different cellular compartments in the tick cell lines and tissues. For example, antibodies against SILK (Figures 2A, B and 3A) and TROSPA (Figures 2C, D and 3B) preferentially recognized intracellular structures and cell membrane, respectively. Anti-SUB antibodies recognized proteins in the nucleus, perinuclear region and cytoplasm of tick cells (Figures 2E-G and 3C). Antibodies against *R. microplus* SUB were tested and showed positive reactions in both *R. microplus* (BME/CTVM2) and *I. scapularis* (IDE8 and ISE6) cell lines.

### Effect of antibodies against tick proteins on tick weight and oviposition and pathogen infection

**Figure 4:**
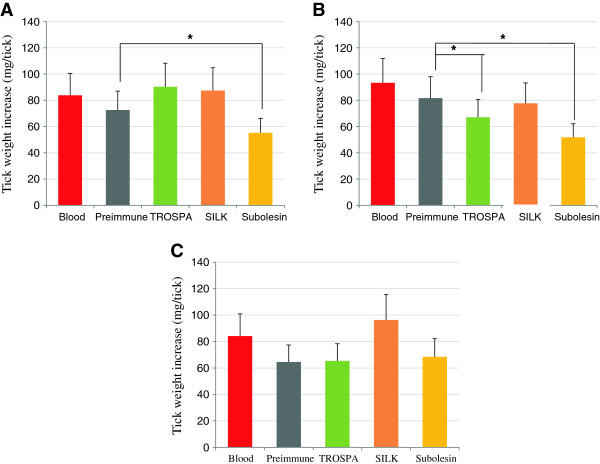
**Effect of antibodies on tick weight. (A)** Ticks capillary-fed on uninfected blood without (Blood) and with preimmune and anti-tick protein IgGs (TROSPA, SILK and Subolesin). **(B)** Ticks capillary-fed on *B. bigemina*-infected blood without (Blood) and with preimmune and anti-tick protein IgGs. **(C)** Ticks capillary fed on *A. marginale*-infected blood without (Blood) and with preimmune and anti-tick protein IgGs. Ticks (N = 15) were weighed before and after capillary feeding, the tick weight increase calculated as final minus initial tick weight, expressed as Ave + S.D. (mg/tick) and compared between the group with preimmune antibodies and the other groups by Student’s t-test (*P < 0.05).

Capillary feeding experiments were conducted to evaluate the effect of antibodies against selected tick proteins on tick weight and oviposition. When ticks were fed with uninfected bovine blood supplemented with antibodies against tick proteins, only the group with anti-SUB IgGs showed a significant reduction in tick weight (24% reduction; P = 0.0003) when compared to ticks fed on blood with preimmune serum (Figure 4A). When ticks were fed on *B. bigemina*-infected blood, only groups with anti-TROSPA and anti-SUB IgGs showed reductions in tick weight, of 18% (P = 0.04) and 37% (P = 0.001) respectively, when compared to ticks fed on blood with preimmune serum (Figure 4B). When ticks were fed on *A. marginale*-infected blood, tick weight was similar between all groups (Figure 4C).

**Figure 5:**
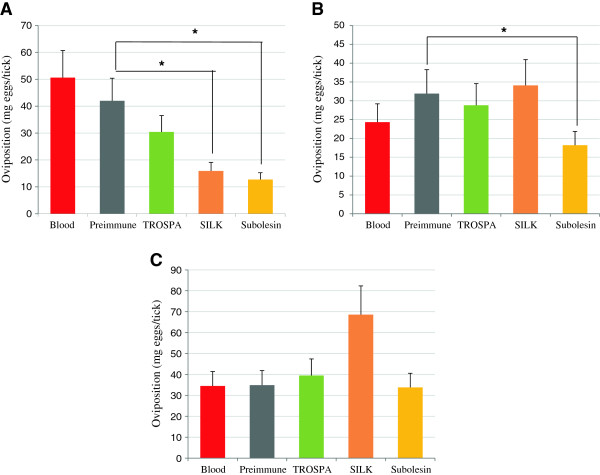
**Effect of antibodies on tick oviposition. (A)** Ticks capillary-fed on uninfected blood without (Blood) and with preimmune and anti-tick protein IgGs (TROSPA, SILK and Subolesin). **(B)** Ticks capillary-fed on *B. bigemina*-infected blood without (Blood) and with preimmune and anti-tick protein IgGs. **(C)** Ticks capillary-fed on *A. marginale*-infected blood without (Blood) and with preimmune and anti-tick protein IgGs. Ticks (N = 5) were incubated for oviposition after feeding, the egg mass weight was determined for each tick, expressed as Ave + S.D. (mg eggs/tick) and compared between the group with preimmune antibodies and the other groups by Student’s t-test (*P < 0.05).

Oviposition was reduced when compared to ticks fed on blood supplemented with preimmune antibodies in the groups fed on uninfected blood with anti-SILK (62% reduction; P = 0.005) and anti-SUB (70% reduction; P = 0.001) antibodies (Figure 5A) and on *B. bigemina*-infected blood with anti-SUB IgGs (43% reduction; P = 0.01) (Figure 5B). No reduction in oviposition was observed in ticks fed on *A. marginale*-infected blood supplemented with antibodies against tick proteins (Figure 5C).

### Effect of antibodies against tick proteins on tick pathogen infection

**Figure 6:**
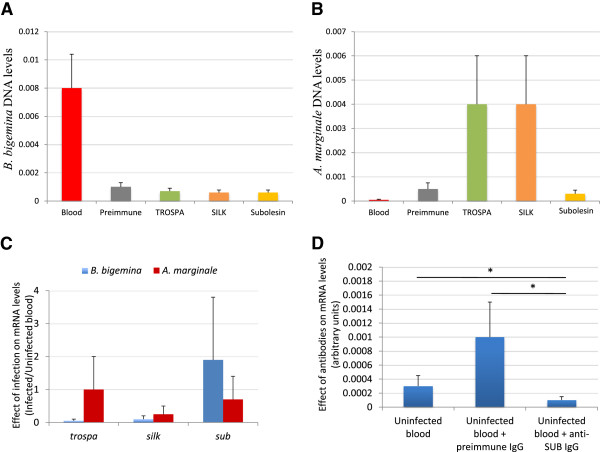
**Effect of antibodies on pathogen infection and gene expression. (A)***B. bigemina* DNA levels were determined by real-time PCR in ticks capillary-fed on *B. bigemina*-infected blood without (Blood) and with preimmune and anti-tick protein IgGs (TROSPA, SILK and Subolesin). The DNA levels were normalized against tick 16S rDNA, shown as Ave + S.D. normalized Ct values (arbitrary units) and compared between the group with preimmune antibodies and the other groups by Student’s t-Test (P > 0.05; N = 10). **(B)***A. marginale* DNA levels were determined by real-time PCR in ticks capillary-fed on *A. marginale*-infected blood without (Blood) and with preimmune and anti-tick protein IgGs. The DNA levels were normalized against tick 16S rDNA, shown as Ave + S.D. normalized Ct values (arbitrary units) and compared between the group with preimmune antibodies and the other groups by Student’s t-Test (P > 0.05; N = 10). **(C)** Effect of *A. marginale* and *B. bigemina* infection on gene expression. The mRNA levels of genes encoding for tick proteins *trospa*, *silk* and *sub* were characterized by real-time RT-PCR in ticks capillary-fed on uninfected and infected blood. The mRNA levels were normalized against tick 16S rRNA, shown as the Ave + S.D. infected/uninfected blood Ct ratio (arbitrary units) and compared between ticks fed on infected and uninfected blood by Student’s t-Test (P > 0.05; N = 10). **(D)** Effect of anti-SUB antibodies on *sub* gene expression. The *sub* mRNA levels were characterized by real-time RT-PCR in ticks capillary-fed on uninfected blood alone or with the addition of preimmune or anti-SUB IgGs. The mRNA levels were normalized against tick 16S rRNA, shown as Ave + S.D. normalized Ct values (arbitrary units) and compared between groups by Student’s t-Test (*p ≤ 0.05; N = 10).

Capillary feeding experiments with antibodies against selected tick proteins were conducted to evaluate the effect on tick infection with *B. bigemina* and *A. marginale*. Pathogen DNA levels in ticks fed on *B. bigemina*-infected blood (Figure 6A) but not in ticks fed on *A. marginale*-infected blood (Figure 6B) were significantly higher when compared to ticks fed on uninfected blood (p < 0.001; data not shown). However, significant differences in pathogen DNA levels at day 3 after feeding were not found between groups fed on blood supplemented with preimmune and immune IgGs (Figure 6A and B).

### Effect of antibodies and pathogen infection on the mRNA levels of genes encoding for tick proteins

The mRNA levels of genes encoding for selected tick proteins were characterized in ticks fed on uninfected and infected blood (Figure 6C). The results showed no difference in gene expression levels between ticks fed on uninfected and infected blood (Figure 6C). Because of the role of SUB as a transcription factor involved in its own regulation, *sub* mRNA levels were characterized in ticks fed on blood supplemented with preimmune and anti-SUB IgGs (Figure 6D). The results showed that anti-SUB IgGs reduced *sub* expression in ticks when compared to ticks fed on uninfected blood (67% reduction; P = 0.05) and blood supplemented with preimmune IgGs (90% reduction; P = 0.01) (Figure 6D).

## Discussion

### SUB

Antibodies against *R. microplus* SUB reacted positively in *R. microplus* BME/CTVM2 cells and tissues and in *I. scapularis* IDE8 and ISE6 cell lines, supporting SUB ubiquitous expression and amino acid sequence conservation in ticks [34]. Vaccination with SUB has shown an effect on the control of *R. microplus* infestations and infection with *A. marginale* and *B. bigemina*[33,35]. In the present study, as in previous experiments with vaccinated cattle [36], anti-SUB antibodies reduced tick weight and oviposition in ticks fed on uninfected blood. However, while tick weight and oviposition were affected by anti-SUB antibodies when ticks were fed on *B. bigemina*-infected blood, no effect was seen with *A. marginale*-infected blood, in contrast to the results of a previous vaccination trial in cattle [33,35]. Additionally, *A. marginale* and *B. bigemina* DNA levels did not differ in ticks fed on infected blood with anti-SUB antibodies when compared to ticks fed on blood supplemented with preimmune serum, again showing differences from the results of the vaccination trial in cattle [33,35]. Additionally, the expression of *sub* has been shown to increase in response to *A. marginale* and *B. bigemina* infection in ticks [33,35]. However, in capillary-fed ticks, *sub* mRNA levels did not increase in response to infection. Nevertheless, the reduction in *sub* mRNA levels in ticks fed on uninfected blood with anti-SUB antibodies suggested that ticks did ingest antibodies in a manner resembling feeding on vaccinated cattle [33,35,36].

SUB is a transcription regulatory factor involved in the control of various tick physiological processes including the immune response to pathogen infection and the expression of genes that are important for pathogen infection and multiplication and for tissue structure and function [34,36-39]. SUB is also involved in the regulation of its own expression through the interaction with NF-kB transcription factors that bind to *sub* core promoter [38]. Therefore, as previously discussed [33,35], the effect of anti-SUB antibodies on *sub* expression and possibly on SUB function could result in reduction in tick weight and oviposition. The proposed model for SUB function as a protective antigen combines the role of this protein in tick immune response and on the control of other genes necessary for pathogen infection [39]. Targeting SUB by vaccination or RNAi reduces tick immunity, thereby increasing pathogen infection levels. However, lower pathogen infection levels may result from the effect of SUB on tissue structure and function and the expression of genes that are important for pathogen infection and multiplication [39]. Similarly to ticks fed on SUB-vaccinated cattle [33,35], the ingestion of less infected blood and interference with pathogen infection and multiplication in ticks capillary-fed on blood with anti-SUB antibodies should have resulted in lower pathogen infection levels. However, reduction in tick pathogen DNA levels were not observed in the present study possibly due to different factors. These factors include (a) tick-to-tick variations in infection levels that require the analysis of a larger number of ticks, (b) parasitemia levels in blood used for capillary feeding (0.7% IE in the present study) that may need to be higher to evidence the effect of the antibodies on pathogen infection, (c) a non-specific effect of the preimmune serum under this experimental conditions and/or (d) differences in pathogen infection mechanisms between *in vivo* tick feeding and *in vitro* capillary feeding. Although Inokuma and Kemp [14] showed that it is possible to infect cattle ticks with *B. bigemina* using capillary feeding with infected blood, Kocan *et al*. [18] demonstrated that the *in vitro* capillary feeding system does not reproduce *in vivo A. marginale* infection in ticks.

### TROSPA

TROSPA was first described in *I. scapularis* as a receptor for *Borrelia burgdorferi,* showing potential as a vaccine antigen to control bacterial infection in ticks [40,41]. In *I. scapularis* and *R annulatus*, *trospa* mRNA levels increased in response to *B. burgdorferi* and *B. bigemina* infection, respectively [11,40]. After gene knockdown by RNAi, *B. bigemina* DNA levels were 83% and 70% lower in *R. annulatus* and *R. microplus*, respectively [11]. Considering TROSPA’s function as a tick receptor for *B. burgdorferi*[40,41], these results suggested that while TROSPA is not involved in tick infestation and oviposition, it might be involved in *B. bigemina* infection and/or multiplication in *R. microplus*. However, in the present study feeding ticks on anti-TROSPA antibodies did not show any effect on pathogen infection, possibly due to some of the factors discussed above for SUB.

### SILK

The flagelliform SILK protein was identified previously in ticks and orb-weaving spider salivary glands [42-44] and was suggested to play a role in *A. marginale* infection and/or multiplication in *R. microplus*[10]. It was shown that *silk* mRNA levels increased in response to *A. marginale* infection of *R. microplus* salivary glands and RNAi experiments showed 74% tick mortality and 63% reduction in *A. marginale* DNA levels after gene knockdown [10]. Additionally, Mulenga *et al*. [45] demonstrated that SILK might be involved in tick attachment. In the present study, ticks fed on uninfected blood with anti-SILK antibodies showed decreased oviposition but similar tick weight when compared to ticks fed on preimmune serum, and anti-SILK antibodies did not show any effect on pathogen infection. Therefore, although previous results suggested a role for SILK in tick**-**host and tick-pathogen interactions, these results were only partially confirmed here.

## Conclusions

The hypothesis behind this experimental approach for the selection of candidate tick protective antigens is that antibodies mediate the main protective mechanism for tick vaccines, something that has been demonstrated for several antigens such as BM86, SUB, SILK and TROSPA for which a direct correlation exists between antibody titers in vaccinated animals and vaccine efficacy [25,35,46-48]. However, if other immune mechanisms are involved in vaccine protection for some antigens, then this experimental approach would not be effective for the selection of protective antigens. The results reported here show that the use of *in vitro* tick capillary feeding has possibilities and limitations for the characterization of candidate tick protective antigens. Ticks fed by capillary feeding ingested antibodies added to the blood meal and the effect of these antibodies was shown on tick weight and oviposition. However, capillary feeding showed limitations in the study of pathogen infection in ticks possibly associated with different factors such as tick-to-tick variation in infection levels, parasitemia levels in blood used for capillary feeding, a non-specific effect of the preimmune serum and/or differences in pathogen infection between *in vivo* tick feeding and *in vitro* capillary feeding, at least for *A. marginale*. Additionally, the results suggested interactions between antigen-specific antibodies and pathogens in the blood meal that could affect the effect on tick weight and oviposition by still unknown mechanisms. Some of these factors could be optimized for each candidate antigen but would add an additional complexity to this approach. Nevertheless, the combination of RNAi functional studies shown in previous work with tick capillary feeding using antibodies against selected proteins involved in tick-pathogen interactions should allow for a better and more efficient selection of candidate vaccine antigens. The use of dsRNA or siRNA for RNAi has the possibility of inducing off-target effects that could mislead the selection of tick candidate protective antigens [49]. However, off-target effects are sequence-specific and, as shown for *sub*, could vary between different molecules [50]. As previously discussed [12], RNAi alone is not a suitable tool for the identification of tick protective antigens, but a tool to integrate with other methods such as capillary feeding using antibodies against selected proteins for the identification and functional characterization of candidate tick protective antigens. In the present study, all three selected antigens, SUB, TROSPA and SILK, showed an effect on tick weight and/or oviposition in ticks fed with uninfected or infected blood containing antigen-specific IgGs. These results together with previous results of RNAi experiments suggest that these proteins are good candidate vaccine antigens for the control of *R. microplus* infestations and infection with *A. marginale* and *B. bigemina*. A recently completed vaccine trial did validate this approach for the selection of candidate tick protective antigens and proved the efficacy of SUB, SILK and TROSPA for the control of *R. microplus* infestations and infection with *A. marginale* and *B. bigemina*[35]. Furthermore, a field trial using the recombinant SUB-MSP1a antigen for vaccination of cattle and sheep showed a reduction in tick infestations and the prevalence for some tick-borne pathogens [51].

## Competing interests

The authors declare that they have no competing interests.

## Authors’ contributions

JF and AD designed the study and performed data analysis; OM, SA and JM performed the tick work. OM, SA, JAM-C, LB-S, PA, RF, SW and JMPL performed the laboratory work. JF wrote the manuscript and all authors edited the manuscript. All authors read and approved the final manuscript.
